# A Pattern of Lipid Profile Among Saudi Adults With Type 1 Diabetes Mellitus in a Tertiary Hospital: A Retrospective Study

**DOI:** 10.7759/cureus.25622

**Published:** 2022-06-03

**Authors:** Awad S Alshahrani, Ahmed R Alibrahim, Mamdouh M Alanazi, Aamir Omair, Muatassem A Alsadhan, Thamer M Alattiah, Fouad A Kanadily, Samaher M Alattiah, Moeber M Mahzari

**Affiliations:** 1 Endocrinology and Metabolism, King Abdulaziz Medical City, Riyadh, SAU; 2 College of Medicine, King Saud Bin Abdulaziz University for Health Sciences College of Medicine, Riyadh, SAU

**Keywords:** saudi arabia, adults, glycemic control, dyslipidemia, type 1 diabetes mellitus

## Abstract

Objective: Dyslipidemia is prevalent in adults living with type 1 diabetes, and it can worsen the presentation of microvascular complications such as retinopathy. This study aims to identify the pattern coupled with the frequency of dyslipidemia in diabetic adults who followed up at different clinics in King Abdulaziz Medical City, Riyadh, and evaluate the associations with demographic and clinical characteristics.

Methods: A cross-sectional, retrospective chart review study of 514 adults with type 1 diabetes was conducted in a tertiary health care facility in the central region of Saudi Arabia. Demographics were retrieved by using the implemented electronic medical records. Fasting lipid profile, glycated hemoglobin (HbA1c), and thyroid-stimulating hormone (TSH) levels were checked for all subjects.

Results: Five hundred and fourteen (514) subjects aged 18-65 years were studied (mean age: 26.1 ± 7.1 years). There were 318 (62%) females in the sample, and their mean age was significantly different from the mean age of males (*p *= 0.01). The mean duration of having diabetes was 12.8 ± 6.9 years. The prevalence of lipid abnormalities included abnormal low-density lipoprotein (LDL) (70%), hypercholesterolemia (23%), abnormal high-density lipoprotein (HDL) (8%), and hypertriglyceridemia (5%). Abnormal HDL was significantly higher in males than in females (*p=*<0.001). There were no statistically significant differences in the prevalence of other lipid abnormalities between the two genders and the age group < or ≥ 25 years. There was no statistically significant difference in the mean of the tested lipids levels between the two genders. One hundred and forty-three (143) (27.8%) patients had more than one abnormal lipid condition. A statistically significant difference was observed in the mean HbA1c between males and females (*p*=0.001). Otherwise, there was no significant association of lipid abnormalities with gender, age, diabetes duration, and weight.

Conclusion: The most prevalent lipid abnormality was high LDL cholesterol. Nearly a third of the tested individuals had more than one lipid abnormality. Furthermore, poor glycemic control was linked to abnormal lipid profiles. Consequently, local programs must aim to screen and intervene early to delay and prevent future severe vascular complications related to non-treated dyslipidemia.

## Introduction

Type 1 Diabetes Mellitus (T1DM) is characterized by the destruction of immune-mediated pancreatic β-cells, leading to absolute insulin deficiency and thus the need for replacement therapy for survival. It is recognized as a serious health problem, supported by the epidemiological data, which reveals a 3%-4% increase in incidence rate per year globally. Furthermore, the age of onset keeps on reducing [1] according to surveys conducted in both developed and developing countries [1,2]. Over the last 40 years, the incidence rate of T1DM has increased in Saudi Arabia [3]. The prevalence of the disease is highest in Riyadh Provinces (126 cases per 100,000) and lowest in Eastern Provinces (48 cases per 100,000) [4].

One of the main aspects of the assessment of T1DM patients is the association of the disease with chronic complications that represent a burden not only to the public healthcare system but also to individuals who cope with them daily. Moreover, it is frequently associated with disabling and life-threatening complications linked to several modifiable risk factors, including an abnormal amount of lipids or Dyslipidemia (DLD) [5].

Lipid abnormalities are common in diabetes mellitus (DM) patients and unquestionably contribute to the increased risk of Atherosclerotic Cardiovascular Disease (ASCVD). The American Diabetes Association (ADA) encourages lipids screening at the time of diagnosis and every five years subsequently for diabetic patients under the age of 40 years, and more often for older patients [6]. DLD is notably seen in people with diabetes irrespective of insulin resistance or deficiency [7]. Low-density lipoprotein (LDL) is the most critical risk factor for ASCVD, in particular Coronary Artery Diseases (CAD), which is the leading cause of death in DM patients [8]. The major classes of DLD are classified according to the Fredrickson phenotype [9], categorized into various defects, and some are familial. It can also result from underlying ‘nonlipid’ causes other than a primary disorder of lipoprotein metabolism. The secondary causes of DLD are DM, cholestatic liver disease (CLD), nephrotic syndrome, chronic kidney disease (CKD), hypothyroidism, obesity, smoking, alcohol consumption, and some medications such as thiazide diuretics, beta-blockers, and hormonal therapy. Lipids that are routinely measured in clinical practice are LDL, high-density lipoprotein (HDL), total cholesterol (Chol), and triglycerides (Trig).

Due to the lack of sufficient data locally in this regard, the study aimed to assess the pattern and frequency of lipids profile in T1DM Saudi adults following up at King Abdulaziz Medical City, Riyadh, (KAMC-RD), and to identify the associations with demographics and clinical characteristics. This would, in turn, aid in better understanding and awareness of the disease that could change our management practices.

## Materials and methods

A single-center retrospective cross-sectional chart review study was conducted to include all adults (≥18 to <65 years of age) with T1DM diagnosis and followed up in different clinics from January 1, 2016, to December 31, 2020, at KAMC-RD, Ministry of National Guard-Health Affairs (MNG-HA), Saudi Arabia. Ethical approval was obtained from the Institutional Review Board of King Abdullah International Medical Research Center (KAIMRC) Approval # NRRC21R/054/02. We retrieved data on subjects’ age, gender, body mass index (BMI), duration of diabetes, glycated hemoglobin (HbA1c), Thyroid-stimulating hormone (TSH), and fasting lipid profile. Subjects without a lipid profile, other DM types, CKD (eGFR <90 mL/min/1.73 m^2^), hypothyroidism, nephrotic syndrome, CLD, smokers, alcohol consumers, non-Saudis, and those on medications like lipid-lowering agents, thiazide diuretics, beta-blocker or hormonal therapy were excluded from the study.

Since pancreatic autoantibodies were not tested among all participants, T1DM was diagnosed clinically by sudden onset of symptoms of diabetes, presenting with diabetic ketoacidosis and markedly elevated HbA1c. This requires insulin from the time of onset among the non-obese and those who did not present insulin resistance signs. All patients were evaluated, and BMI was classified as per WHO criteria [10]: low weight, normal weight, overweight, and obesity. Furthermore, lipid level was taken after a minimum fasting period of 8-12 hours.

Lipid abnormalities were defined based on the modified ADA criteria [11] and the Third Report of the National Cholesterol Education Program (NCEP III) [12]. Lipids were categorized into normal and abnormal levels. Abnormal levels were defined as total Cholesterol >5.18 mmol/L, triglyceride >1.7 mmol/L, HDL <1.55 mmol/L, and LDL >2.59 mmol/L. Diagnosis of dyslipidemia was made when one or more lipid values were abnormal. HbA1c was classified as optimal if <7%, suboptimal if 7%-10%, and poor if >10%. Moreover, the normal TSH range was 0.35-4.94 mlU/L. Subjects were also classified into < 25 years and ≥ 25 years of age groups. The diabetes duration was classified into ≤ 10 years and > 10 years.

The data was entered into a spreadsheet and analyzed using IBM Corp. Released 2016. IBM SPSS Statistics for Windows, Version 24.0. Armonk, NY: IBM Corp. The categorical variables were presented as frequency and percentages, while the numerical variables were presented as mean ± standard deviation. The independent samples t-test was used to compare the numerical variables between two groups, while the Chi-Square test was tested to compare the categorical variables. A *p*-value of < 0.05 was considered significantly different for all the statistical tests.

## Results

A total of 764 subjects were reviewed. Of those, 514 were included and analyzed after meeting the inclusion criteria. The mean age was 26.1 ± 7.1 years, and their mean duration of having diabetes was 12.8 ± 6.9 years. There were 318 (62%) females in the sample, and their mean age of 26.7 ± 7.1 years was significantly different from the mean age of 25.1 ± 7.1 for males (*p* = 0.01).

Demographic and metabolic parameters by gender are shown in Table 1. The mean age was 26.1 ± 7.1 years, and BMI was 25.7 ± 5.6 kg/m2, respectively. Females had a statically higher BMI (*p*<0.001). They also had a significantly higher DM duration at 13.7 years (*p*<0.001). Males had a significantly lower HDL level at 1.3 mmol/L compared to females at 1.5 mmol/L (*p*=<0.001). Additionally, TSH was statistically significant between the two groups (*p*<0.001). No significant difference was found for HbA1c, LDL, triglycerides or total cholesterol between males and females. The mean levels of LDL, HDL, triglycerides, and total cholesterol of the study population were 3.0 ± 0.8, 1.4 ± 0.3, 0.91 ± 0.52, and 4.7 ± 0.9, respectively.

**Table 1:** Demographic and clinical parameters of the subjects given as Mean ± SD * Five subjects (four males and one female) are missing for Triglyceride.

Characteristics	Total (n=514)	Male (n=196)	Female (n=318)	P-value
Age, years	26.1 ± 7.1	25.1 ± 7.1	26.7 ± 7.1	.01
BMI, kg/m2	25.7 ± 5.6	24.4 ± 5	26.5 ± 5.9	<0.001
Diabetes duration, years	12.8 ± 6.9	11.3 ± 6.6	13.7 ± 6.9	<0.001
HbA1c, %	8.7 ± 1.7	8.8 ± 1.9	8.7 ± 1.7	.55
TSH, mlU/L	2.1 ± 1.1	1.9 ± 0.99	2.3 ± 1.2	<0.001
LDL, mmol/L	3.0 ± 0.8	3.1 ± 0.9	3.0 ± 0.8	.70
HDL, mmol/L	1.4 ± 0.3	1.3 ± 0.3	1.5 ± 0.3	<0.001
Triglycerides*, mmol/L	0.91 ± 0.52	0.98 ± 0.61	0.87 ± 0.45	.04
Cholesterol, mmol/L	4.7 ± 0.9	4.6 ± 0.9	4.7 ± 0.8	.08

Table 2 shows the prevalence of different lipid abnormalities by gender group. The commonest dyslipidemia was a high LDL level in 358 (70%) patients. Low HDL was significantly higher in males (*p*=<0.001). Otherwise, there was no difference in the prevalence of other lipid abnormalities between the genders.

**Table 2:** Prevalence of lipid abnormalities by gender * Five subjects (four males and one female) are missing for Triglycerides.

Lipid type	Total n (%)	Male n=196 (%)	Female n=318 (%)	P-value
LDL				0.77
Normal	156 (30%)	58 (30%)	98 (31%)	
Abnormal	358 (70%)	138 (70%)	220 (69%)	
HDL				<0.001
Normal	471 (92%)	166 (85%)	305 (96%)	
Abnormal	43 (8%)	30 (15%)	13 (4%)	
Triglycerides *				0.053
Normal	484 (95%)	178 (93%)	306 (97%)	
Abnormal	25 (5%)	14 (7%)	11 (3%)	
Cholesterol				0.13
Normal	396 (77%)	158 (81%)	238 (75%)	
Abnormal	118 (23%)	38 (19%)	80 (25%)	

The prevalence of different lipid abnormalities by age group is shown in Table 3. There was no statistical difference in lipid abnormalities in the age group < and ≥ 25 years.

**Table 3:** Prevalence of lipid abnormalities by age group * 5 subjects are missing from the <25 years group.

Lipid type	Total n (%) (N=514)	< 25 years n=263 (%)	≥ 25 years n=251 (%)	p-value
LDL				0.68
Normal	156 (30%)	82 (31%)	74 (29%)	
Abnormal	358 (70%)	181 (69%)	177 (71%)	
HDL				>0.999
Normal	471 (91.6%)	241 (92%)	230 (92%)	
Abnormal	43 (8.4%)	22 (8%)	21 (8%)	
Triglycerides *				0.17
Normal	484 (95%)	242 (94%)	242 (96%)	
Abnormal	25 (5%)	16 (6%)	9 (4%)	
Cholesterol				0.73
Normal	396 (77%)	201 (76%)	195 (78%)	
Abnormal	118 (23%)	62 (24%)	56 (22%)	

Differences in the mean variables between subjects with <2 and ≥2 dyslipidemia are shown in Table 4. There was a significant difference in the mean HbA1c among both groups (*p*=0.004).

**Table 4:** Characteristics of subjects based on number of lipid abnormalities

Variable	< 2 lipid abnormalities (n=365)	≥ 2 lipid abnormalities (n=143)	p-value
Age (years)	26.2 ± 7.0	26.1 ± 7.0	0.96
HbA1c (%)	8.59 ± 1.69	9.08 ± 1.81	0.004
Mean duration of DM (years)	13.0 ± 6.8	12.5 ± 7.2	0.49
Mean BMI (kg/m^2^)	25.57 ± 5.59	26.18 ± 5.82	0.27
Mean TSH (mlU/L)	2.11 ± 1.10	2.18 ± 1.13	0.52

Table 5 displays the difference in proportion by age and other characteristics amongst subjects with ≥2 and 1 or no dyslipidemia. More females than males had dyslipidemia. Furthermore, dyslipidemia was more prevalent in < 25-year-olds, those who had diabetes for a duration of ≤ 10 years, and people with obesity. Nonetheless, these results were not statistically significant, apart from the mean HbA1c, which significantly differed in both groups (*p*=0.001). Various studies are looking at the abnormal lipid levels in diabetic patients; an important factor for the differences in data (%) presented (Appendix 1) is due to the variability in reference ranges. Appendix 2 shows the reasons for subjects' exclusion.

**Table 5:** Relationship between clinical characteristics and dyslipidemia ** One subject had a missing value for the duration of diabetes in the <2 lipid abnormalities category.

Parameters	< 2 lipid abnormalities (n=366)	≥ 2 lipid abnormalities (n=143)	P-value
Gender			0.83
Male	137 (71%)	55 (29%)	
Female	229 (72%)	88 (28%)	
Age			0.77
< 25 years	187 (72%)	71 (28%)	
≥ 25 years	179 (71%)	72 (29%)	
Duration of diabetes** n=365			0.71
≤ 10 years	144 (71%)	59 (29%)	
> 10 years	221 (72%)	84 (28%)	
HbA1c			0.001
<7%	41 (73%)	15 (27%)	
7-10%	269 (76%)	86 (24%)	
>10%	56 (57%)	42 (43%)	
Weight category			0.64
Underweight	30 (75%)	10 (25%)	
Normal weight	156 (72%)	61 (28%)	
Overweight	105 (74%)	36 (26%)	
Obese	75 (68%)	36 (32%)	

## Discussion

The present study discloses a high prevalence of dyslipidemia that reaches 70%. This high rate of dyslipidemia is consistent with other studies [13,14]. Many studies have shown similar findings in children and adolescents age groups [15-18]. In their study on dyslipidemia in Bangladeshi adults with T1DM, Zabeen et al. [13] indicated that 50% of the subjects had dyslipidemia. Equivalently, Bhambhani et al. [14] reported a prevalence of 19% of lipid abnormalities upon evaluating selected adults with insulin-dependent diabetes.

However, distinct prevalence rates of dyslipidemia have also been reported [19,20]. Pérez et al. [19] found a prevalence of dyslipidemia of 20% in Spanish adults, while Demirel et al. [20] found a prevalence of dyslipidemia of 30% in Turkish adolescents with TIDM. The differences in data presented by different studies are due to several factors such as differences in the reference ranges, targeted age, diabetes duration, treatment regimens, and glycemic control.

A study investigating cardiovascular risk factors in more than 11,000 children and adolescents (aged from 2 to 18 years) with T1DM was conducted in the United States. Redondo et al. [21] reported a 3.8% prevalence of dyslipidemia, attributing the low prevalence to the fact that the majority of the subjects were neither young nor obese.

High LDL (70%) was the commonest dyslipidemia detected in our subjects, which is by far higher than the numbers from various studies [13-15].

Hypercholesterolemia has been recorded as the most prevalent type of dyslipidemia in various studies [18,20,22]. In Egypt, Mona et al. [23] reported high LDL and low HDL as the commonest types of dyslipidemia and reported hypertriglyceridemia in less than 5% of subjects. On the contrary, Bulut et al. [24] reported that hypercholesterolemia was the most frequent type of dyslipidemia, whereas hypertriglyceridemia was seen in only 12.9% of subjects.

Nevertheless, in a report of lipid profiles from Nigeria, Jaja et al. [25] reported hypertriglyceridemia as the commonest dyslipidemia in T1DM participants (mean age: 14.94 ± 3.59 years). The variance of dyslipidemia prevalence might be due to dietary habits variations, glycemic control, age, and coexistence of other medical conditions.

The dietary pattern was not precisely studied in this study, though it is well known that fats and carbohydrates are the main components of Saudi’s diet. Unfortunately, poor glycemic control and suboptimal insulin therapy are also common in Saudi DM patients [26,27], which can help explain the prevalence of high LDL compared to other studies.

It is no doubt that LDL is the foremost risk factor for ASCVD, such as CAD [28]. LDL is one of the five major groups of lipoprotein that transport all fat molecules around the body in the extracellular water [29]. LDL particles are formed when triglycerides are separated from very-low-density lipoprotein (VLDL) using the lipoprotein lipase enzyme (LPL). Later, they become denser and smaller (i.e., same protein transport shells with fewer fat molecules), composing higher cholesterol esters [30]. LDL has been associated with the evolution of atherosclerosis and blockage of the artery lumen due to its ability to carry cholesterol into smaller vessels [31]. LDL is also crucial for carrying lipids that keep us alive, like those responsible for the innate immune system [32].

According to the ADA guidelines [6] on dyslipidemia, pharmacological therapy is recommended if the LDL level is ≥ 3.35 mmol/l (130 mg/dl). The ideal LDL cholesterol level is < 2.60 mmol/l (<100 mg/dl). Based on that, 70% of our subjects (who had high LDL levels) are considered to require intervention.

The mean values of most lipids were observed to be higher in males than in females. Both genders have almost the same percentage of more than one abnormal dyslipidemia (29% in males and 28% in females). Comparable findings were noted in Turkey by Bulut et al. [24], who found that both males (26.1%) and females (26.2%) had almost similar prevalence of dyslipidemia. On the other side, females were also observed in other studies to have a higher prevalence of dyslipidemia [17,33]. According to Homma et al. [17], females had a higher prevalence of dyslipidemia (87%). Similarly, Franca et al. [33] reported equivalent outcomes of dyslipidemia between females (34.7%) and males (25.3%).

One theory behind the reason for the higher dyslipidemia tendency in females has been mentioned by Pérez et al. [19], who proposed that diabetes in women has a most outstanding impact on cardiovascular risk. They also suggested a higher atherogenic risk among them despite well-controlled diabetes.

In our study, the mean age was higher for females than males (26.7 vs. 25.1 ± 7.1 years), as well as a higher mean BMI (26.5 ± 5.9 vs. 24.4 ± 5 kg/m2) and nearly a comparable HbA1c level between the two genders (8.8 ± 1.9 vs. 8.7 ± 1.7%). In this study, there was a significant relationship between higher HbA1c levels and dyslipidemia. Otherwise, there was no significant relationship between other parameters (genders, age, duration of diabetes, and weight) and dyslipidemia.

Nevertheless, numerous studies have represented the relationship between glycemic control (expressed by HbA1c) and dyslipidemia [16,33,34]. In ours, females with a higher mean HbA1c and a higher prevalence of dyslipidemia were identified. Over and above, subjects with optimal HbA1c of <7% revealed a statistically significant difference regarding dyslipidemia compared with those with poor HbA1c of >10%.

Limitations

This study did not account for micro- and macro-vascular complications and autoimmune comorbidities, owing to the institution’s financial burden. Other limitations were the lack of details on caloric and nutrient contents of our diets as preparation methods differ, which sequentially affected nutrient content. Besides examinations and vital signs, physical activity and lifestyle were not discussed for patients enrolled in the study. A case-control and subsequent prospective studies (including a larger sample) will be necessary to draw more practical conclusions on dyslipidemia in adults living with T1DM in Saudi Arabia.

## Conclusions

The most prevalent lipid abnormality was high LDL cholesterol. Around a third of the tested individuals had more than one lipid abnormality. Poor glycemic control was clearly linked to abnormal lipids profile. Consequently, local programs must aim to screen and intervene early to delay and prevent future serious vascular complications related to non-treated dyslipidemia.

## Appendices

Appendix 1

**Table 6:** Pattern of lipid profile in Type 1 Diabetes Mellitus (comparison between different studies) T1DM: Type-1 Diabetes Mellitus, DLD: Dyslipidemia, LDL: low-density lipoproteins, HDL: High-density lipoproteins, TC: Total cholesterol, TG: Triglycerides.

No.	Authors/Years/Reference	Study design	Country	Sample size	DLD %	Patient characteristics
1	Alshahrani A et al. 2021	Retrospective cross-sectional study	Saudi Arabia	514	70% abnormal LDL - 8% abnormal HDL - 5% abnormal TG - 23% abnormal TC	T1DM/ Mean age: 26.1 ± 7.1 years
2	Abed E et al. 2019 [35]	Retrospective cross-sectional study	USA	129	34.88% abnormal LDL - 27.90% abnormal HDL - 27.13% abnormal TG - 20.93 % abnormal TC	T1DM/ Mean age: 17.59 ± 2.30 years
3	Zabeen B et al. 2018 [13]	Prospective cross-sectional study	Bangladesh	422	23% abnormal LDL - 29% abnormal HDL - 50% abnormal TG - 33% abnormal TC	T1DM/ Mean age: 47.32 ± 8.62 years
4	Bhambhani G et al. 2015 [14]	Retrospective cross-sectional study	India	100	19% abnormal LDL - 5% abnormal HDL - 5% abnormal TG - 12% abnormal TC	T1DM/ Mean age: 32.6 years
5	Perez et al. 2000 [19]	Prospective cross-sectional study	Spain	334	16%% abnormal LDL - 20% abnormal HDL - 5% abnormal TG	T1DM/ Mean age: 31.3 ± 10.2 years

Appendix 2

**Table 7:** Reasons of exclusion

Reason	Number of subjects
Other diabetes types: Neonatal DM	1
Latent autoimmune diabetes in adults (LADA)	4
Type 2 diabetes mellitus (T2DM)	63
Nephrotic syndrome	3
Cholestatic liver disease (CLD)	7
Non-saudi	6
On hormonal therapy	11
Smoking	15
No lipid profile	25
Chronic kidney disease (CKD)	33
Hypothyroidism	36
On statin	46
Total	250

## References

[1] Tuomilehto J (2013). The emerging global epidemic of type 1 diabetes. Curr Diab Rep.

[2] Al Shaikh A, Al Zahrani AM (2016). Impact of vitamin D status on cardiometabolic complications among children and adolescents with type 1 diabetes mellitus. J Clin Res Pediatr Endocrinol.

[3] Cherian MP, Al-Kanani KA, Al Qahtani SS (2010). The rising incidence of type 1 diabetes mellitus and the role of environmental factors--three decade experience in a primary care health center in Saudi Arabia. J Pediatr Endocrinol Metab.

[4] Al-Herbish AS, El-Mouzan MI, Al-Salloum AA, Al-Qurachi MM, Al-Omar AA (2008). Prevalence of type 1 diabetes mellitus in Saudi Arabian children and adolescents. Saudi Med J.

[5] Elham Elham, N. N., & Flora, M. S (2013). Pattern of lipid profile among Type 2 diabetic patients. Ibrah Med Col J.

[6] American Diabetes Association (2020). Cardiovascular disease and risk management: standards of medical care in Diabetes-2020. Diab Cr.

[7] Taskinen MR (2003). Diabetic dyslipidaemia: from basic research to clinical practice. Diabetologia.

[8] Lee YB, Han K, Kim B (2019). Risk of early mortality and cardiovascular disease in type 1 diabetes: a comparison with type 2 diabetes, a nationwide study. Cardiovasc Diabetol.

[9] Fredrickson DS (1971). An international classification of hyperlipidemias and hyperlipoproteinemias. Ann Intern Med.

[10] Feingold KR, Anawalt B, Boyce A (2015). Dietary treatment of obesity. Endotext.

[11] Haffner SM (2003). Management of dyslipidemia in adults with diabetes. Diabetes Care.

[12] (2001). Executive summary of the third report of the National Cholesterol Education Program (NCEP) expert panel on detection, evaluation, and treatment of high blood cholesterol in adults (Adult treatment panel III). JAMA.

[13] Zabeen B, Balsa AM, Islam N, Parveen M, Nahar J, Azad K (2018). Lipid profile in relation to glycemic control in Type 1 diabetes children and adolescents in Bangladesh. Indian J Endocrinol Metab.

[14] Bhambhani Bhambhani, G. G., Bhambhani Bhambhani, R. R., & Thakor, N N (2017). Lipid profile of patients with diabetes mellitus: a cross sectional study. International Journal of Research in Medical Sciences.

[15] Al-Agha AE, Alafif MM, Abd-Elhameed IA (2015). Glycemic control, complications, and associated autoimmune diseases in children and adolescents with type 1 diabetes in Jeddah, Saudi Arabia. Saudi Med J.

[16] Guy J, Ogden L, Wadwa RP (2009). Lipid and lipoprotein profiles in youth with and without type 1 diabetes: the SEARCH for diabetes in youth case-control study. Diabetes Care.

[17] Homma TK, Endo CM, Saruhashi T, Mori AP, Noronha RM, Monte O, Calliari LE (2015). Dyslipidemia in young patients with type 1 diabetes mellitus. Arch Endocrinol Metab.

[18] Rahma S, Rashid JA, Farage AH (2006). The significance of lipid abnormalities in children with insulin-dependant diabetes mellitus. Iraqi Post Med J.

[19] Pérez A, Wägner AM, Carreras G (2000). Prevalence and phenotypic distribution of dyslipidemia in type 1 diabetes mellitus: effect of glycemic control. Arch Intern Med.

[20] Demirel F, Tepe D, Kara O, Esen I (2013). Microvascular complications in adolescents with type 1 diabetes mellitus. J Clin Res Pediatr Endocrinol.

[21] Redondo MJ, Foster NC, Libman IM (2016). Prevalence of cardiovascular risk factors in youth with type 1 diabetes and elevated body mass index. Acta Diabetol.

[22] Polkowska A, Głowińska-Olszewska B, Tobiaszewska M, Bossowski A (2015). [Risk factors for cardiovascular disease in children with type 1 diabetes in 2000-2010 in Podlasie Province]. Pediatr Endocrinol Diabetes Metab.

[23] Mona HM, Sahar SA, Hend SM, Nanees AW (2015). Dyslipidemia in type 1 diabetes mellitus. Relation to diabetes duration, glycemic control, body habitus, dietary intake and other epidemiological risk factors. Egyptian Pediatric As- soc Gazette.

[24] Bulut T, Demirel F, Metin A (2017). The prevalence of dyslipidemia and associated factors in children and adolescents with type 1 diabetes. J Pediatr Endocrinol Metab.

[25] Jaja T, C C, Yarhere I, E E (2019). Dyslipidaemia in Nigerian children and adolescents with diabetes mellitus: prevalence and associated risk factors. Int J Diabetes Metab.

[26] Al Zahrani AM, Al Shaikh A (2019). Glycemic control in children and youth with type 1 diabetes mellitus in Saudi Arabia. Clin Med Insi Endo Diab.

[27] Aljabri KS, Bokhari SA (2013). Glycemic control of patients with type 1 diabetes mellitus in Saudi community. J Diabetes Metab.

[28] Carson JA, Lichtenstein AH, Anderson CA (2020). Dietary cholesterol and cardiovascular risk: a science advisory from the American Heart Association. Circulation.

[29] (2017). Centers for Disease Control and Prevention: LDL and HDL cholesterol: "bad" and "good" cholesterol. http://www.cdc.gov/cholesterol/ldl_hdl.htm.

[30] Linton MRF, Yancey PG, Davies SS (2019). The role of lipids and lipoproteins in atherosclerosis. Endotext [Internet].

[31] Singh V, Sharma R, Kumar A, Deedwania P (2010). Low high-density lipoprotein cholesterol: current status and future strategies for management. Vasc Health Risk Manag.

[32] Peterson MM, Mack JL, Hall PR (2008). Apolipoprotein B Is an innate barrier against invasive Staphylococcus aureus infection. Cell Host Microbe.

[33] Giuffrida FM, Guedes AD, Rocco ER (2012). Heterogeneous behavior of lipids according to HbA1c levels undermines the plausibility of metabolic syndrome in type 1 diabetes: data from a nationwide multicenter survey. Cardiovasc Diabetol.

[34] Chowdhury S (2015). Puberty and type 1 diabetes. Indian J Endocrinol Metab.

[35] Abed E, LaBarbera B, Dvorak J, Zhang Y, Beck J, Talsania M (2019). Prevalence of dyslipidemia and factors affecting dyslipidemia in young adults with type 1 diabetes: evaluation of statin prescribing. J Pediatr Endocrinol Metab.

